# Sensitivity-Improved Strain Sensor over a Large Range of Temperatures Using an Etched and Regenerated Fiber Bragg Grating

**DOI:** 10.3390/s141018575

**Published:** 2014-10-08

**Authors:** Yupeng Wang, Xueguang Qiao, Hangzhou Yang, Dan Su, Ling Li, Tuan Guo

**Affiliations:** 1 School of Physics, Northwest University, Xi'an, Shaanxi 710069, China; E-Mails: xgqiao@nwu.edu.cn (X.Q.); yanghz@nwu.edu.cn (H.Y.); dansuf@163.com (D.S.); liling559@163.com (L.L.); 2 Institute of Photonics Technology, Jinan University, Guangzhou 510632, China

**Keywords:** etched and regenerated fiber gratings, strain sensor, high temperature

## Abstract

A sensitivity-improved fiber-optic strain sensor using an etched and regenerated fiber Bragg grating (ER-FBG) suitable for a large range of temperature measurements has been proposed and experimentally demonstrated. The process of chemical etching (from 125 μm to 60 μm) provides regenerated gratings (at a temperature of 680 °C) with a stronger reflective intensity (from 43.7% to 69.8%), together with an improved and linear strain sensitivity (from 0.9 pm/με to 4.5 pm/με) over a large temperature range (from room temperature to 800 °C), making it a useful strain sensor for high temperature environments.

## 1. Introduction

Diverse fiber Bragg gratings (FBGs) have been proposed and widely used as fiber-optic sensors to measure strain, displacement, refractive index and temperature due to their intrinsic advantages [1–3]. Among them, strain measurement must be performed in many high temperature environments, such as space plane engines, high temperature pipelines in the oil and gas industry, *etc.* To improve the temperature resistance of FBG for strain sensing, many techniques have been put forward in the past few years. Bragg gratings inscribed in Er-Sn-codoped germanosilicate fiber were employed to simultaneously measure the strain range of 0–1150 με at temperatures up to 500 °C with a strain sensitivity of 1.08 pm/με [4]. An alternative approach has been demonstrated by writing the grating in Sb-Er-Ge-codoped fiber, which enhances the temperature and strain sustainability to 600 °C and 2000 με, respectively, and a strain sensitivity of 1.15 pm/με is achieved [5]. Li *et al.* have reported the temperature response of chemical composite gratings over a temperature range from 24 °C to 900 °C and the strain response at a maximum applied strain of 1000 με, with a strain sensitivity of 1.1 pm/με at each temperature [6].

From the reports above, a strain sensor based on a bare FBG has a strain sensitivity of around 1 pm/με, which results in a low detection resolution. Conventionally, two approaches have been employed to enhance the strain sensitivity: one is recoating the fiber sensor with a layer of metal, the other is chemically etching the fiber sensor. A metal-packaged regenerated fiber Bragg grating (RFBG) using titanium–silver magnetron sputtering and nickel electroplating was developed for strain measurement [7], in which the strain sensitivity is 1.7 pm/με at temperatures ranging from room temperature to 400 °C. Lyons *et al.* presented a method involving chemically etched cladding to study the characteristic of FBGs and propose a tunable FBG filter, which has a repeated wavelength tuning range of 9 nm [8]. A FBG with a diameter of only 12 μm prepared through wet chemical etching has been employed to measure strain. The results show that it has ultra-high strain sensitivity up to 104.1 pm/με in the strain range only 0–100 με and temperature up to 65 °C [9].

Compared with the metal coating technique, chemical etching is a much easier and cost effective method to enhance the strain sensitivity of fiber sensor. Furthermore, RFBG has been used as an ideal candidate for operating at ultra-high temperatures [10–12]. In this work, we demonstrate an approach to enhance the reflective intensity of RFBG, improve its strain sensitivity and enlarge its temperature measurement range. The chemical etching has been performed on the seed gratings to get FBGs with different diameters. A standard thermal treatment technique is used to activate these seed gratings and obtain etched and regenerated fiber Bragg gratings (ER-FBGs). The results show the reflective intensity of ER-FBG can be effectively enhanced by reducing the fiber diameter. Meanwhile, an improved strain sensitivity (from 0.9 pm/με to 4.5 pm/με) over a large range of temperature measurements (from room temperature to 800 °C) have been experimentally achieved, making it a good candidate for strain sensors in ultrahigh temperature environments.

## 2. Fabrication and Experiment

The seed gratings (2.0 cm in length) are written in B/Ge codoped photosensitive fiber (PS1250/1500, Fibercore Ltd., Hampshire, UK). A continuous wave (CW) frequency-doubled argon ion laser with an operation wavelength of 244 nm and average output power of 28 mW is used. The grating is inscribed with well-established phase mask method. A phase mask with pitch period of 1072.6 nm is used for seed grating inscription. It should be noted that we use a laser beam scanning method (along fiber axial direction) to inscribe the grating. Therefore, the length of the grating is essentially dominated by the length of the phase mask (2.0 cm is used here).The fibers used (initial cladding diameter of 125 μm) are of 10 mol% of GeO_2_ and 14–18 mol% of B_2_O_3_ and with core-cladding refractive index difference of 0.5%. Before grating inscription, the fibers are hydrogen loaded under a pressure of 11.3 MPa for 10 days at room temperature. The fabricated seed gratings are stored in a drying oven (50 °C) for two days to diffuse out the hydrogen. The grating strength then decays and stabilizes at 26 dB.

**Figure 1.**
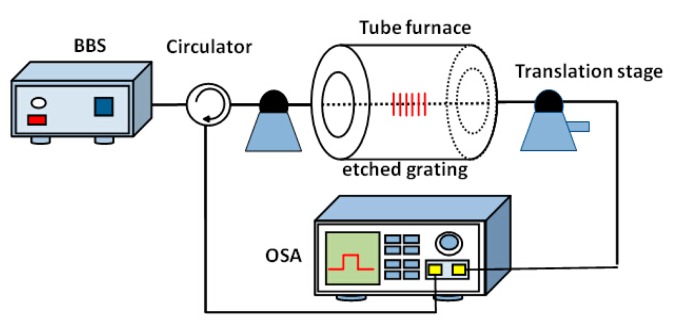
Schematic diagram of the experimental setup.

The etched FBGs with uniform fiber diameter are achieved by immersing them in HF acid solution (40% in water). During etching, the fiber diameter is measured by a microscope. As a result, the FBGs with different diameters of 125 μm, 90 μm and 60 μm are acquired, respectively. The fiber diameter of 60 μm is selected as a case, which increases the strain sensitivity without changing the FBG temperature and strain properties for large ranges of temperature and strain measurement. The experimental setup for thermal regeneration of FBG and the strain response at high temperature for etched RFBG is shown in Figure 1. Amplified spontaneous emission (ASE) from an erbium doped fiber amplifier (EDFA) is used as a broadband light source (BBS), which is connected through a circulator to the etched FBG. The etched FBG is positioned (stain free) in the tube furnace for isothermal annealing process. Both transmission and reflection spectra of the etched gratings are measured using an optical spectrum analyzer (OSA) with a wavelength resolution of 0.02 nm. Thermal regeneration is then applied for the seed gratings through a subsequent annealing process. To characterize the response of the regenerated gratings with longitudinally applied strain, the both sides of RFBG are fixed by using epoxy, in which one side is fastened on a micro-displacement stage with a displacement resolution of 0.01 mm. The fiber is then stretched by a calibrated micrometer and the measurements of both ΔL and L are taken. To make sure the grating provides a linear strain response for strain measurement, we pre-strained the grating before it is fixed (glued). The initial tension (pre-strain) can be measured by monitoring the wavelength shift of FBG before and after the grating is fixed, and the initial strain value is 0 uε. A displacement step of 0.1 mm applied to 1.0 m fiber is equal to a strain step of 100 με. Each increment and the corresponding λ_B_ are recorded, simultaneously.

**Figure 2.**
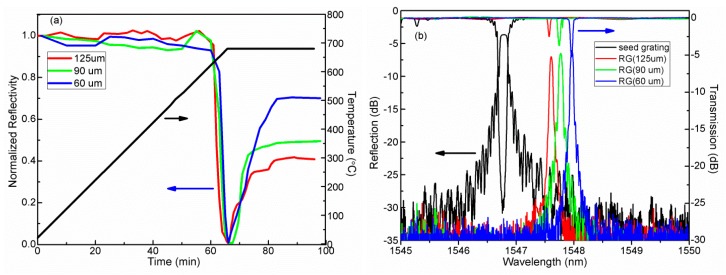
(**a**) Evolution of etched gratings reflectivity during thermal regeneration. (**b**) The reflection and transmission spectrum of seed (black line) and regenerated gratings, namely 125 μm (red curve), 90 μm (green curve) and 60 μm (blue curve) respectively.

Figure 2a shows the measured dynamics of grating regeneration for different diameter fibers. The color curves represent the grating regeneration dynamics of three grating samples with different diameters, while the black curve represents the temperature change at the corresponding time. The heating temperature is ramping at 10 °C/min to 680 °C at which the seed grating is totally immersed into the noise level. The fiber is then held at 680 °C for 30 min to activate the regeneration behavior of the grating. After that, the heating temperature is passively cooled down to room temperature. Figure 2b shows the reflection and transmission spectra of the seed gratings at room temperature (black curves) and their RFBGs with different diameters, respectively.

## 3. Results and Discussion

Clearly, according to Figure 2, we get reflectivity-improved regenerated FBGs via cladding etching, which correspond to reflectivity of 43.7% (diameter of 125 μm), 55.3% (diameter of 90 μm) and 69.8% (diameter of 60 μm). This is because the condition of grating regeneration appears to be strongly correlated with differences in glass relaxation between the cladding and core [13]. The process of reducing the cladding diameter helps to accentuate this difference, and leads to stronger grating regeneration.

**Figure 3.**
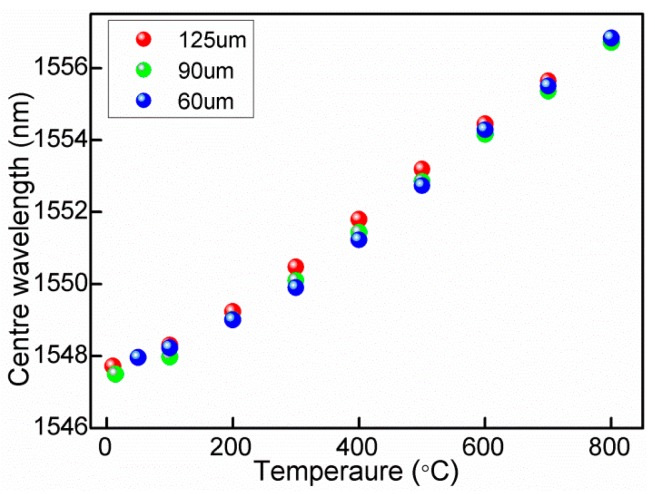
Wavelength shift of RFBGs with different diameters versus temperature range from 25 to 800 °C.

The temperature response of the RFBGs is tested from 25 °C to 800 °C. The temperature of the tube furnace is increased from room temperature to 800 °C by a controllable stepwise ramp of 10 °C/min. To avoid temperature fluctuations during the measurement process, the temperature of the tube furnace is kept constant for 20 min at each step of 100 °C to ensure uniform distribution before each recording. Figure 3 shows the temperature responses of three RFBGs with different diameters for increasing the temperature. The temperature sensitivities of 12.0 pm/°C, 12.1 pm/°C, and 12.2 pm/°C are obtained for fiber diameters of 125, 90 and 60 μm, respectively. The temperature sensitivities are almost constant with reducing the RFBG's diameter, which may be due to the fact that thermo-optic coefficient is consistent for that different diameters.

For a normal fiber grating, the applied strain will induce the shift of central wavelength due to co-effects of the change in grating period, and the shift in core index of refraction caused by the photo elastic effect. The strain effect of grating is given as:

(1) $$\frac{\Delta \lambda_{B}}{\lambda_{B}}=(1-p_{e})ɛ$$

where ε is the applied strain in the axial fiber and *p_e_* is the effective elastic-optic coefficient given by:

(2) $$p_{e}=\frac{{n_{eff}}^{2}}{2}[p_{12}-\upsilon (p_{11}+p_{12})]$$

where *p*_11_ and *p*_12_ are the components of the silica strain-optic tensor, and *υ* is Poisson's ratio. For typical silica optical fibers, *p*_11_ = 0.121, *p*_12_ = 0.270, *υ* = 0.17 and *n_eff_* = 1.456, we can present a grating strain sensitivity coefficient *K_ε_*, *K_ε_* = 1 – *p_e_* = 0.784. For a FBG with a center wavelength of 1550 nm, the expected strain sensitivity is approximately 1.2 pm/με. By reducing the cross sectional area of a fiber, etching can induce a significant reduction in the force required to produce a given strain [8]. It offers the possibility of improving the strain sensitivity for etched cladding FBG applied the same strain, compared with standard 125 μm FBG.

**Figure 4.**
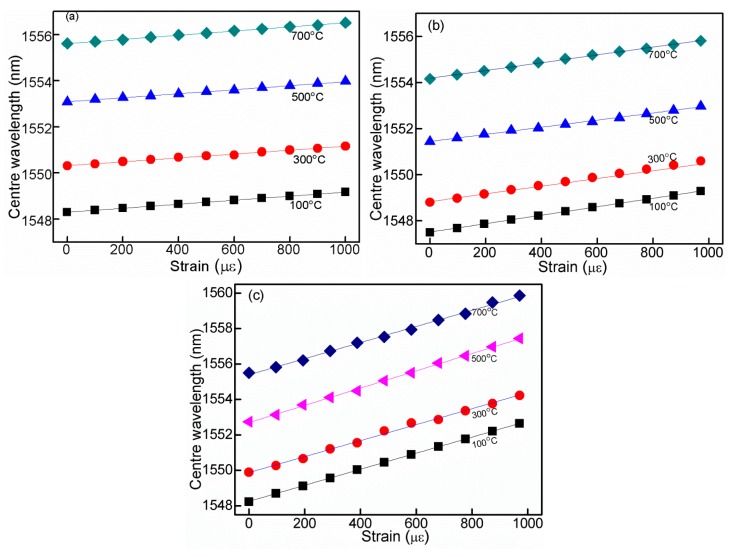
Wavelength shift of ER-FBGs versus axial strain at various temperatures: RFBG diameters of (**a**) 125 μm, (**b**) 90 μm, and (**c**) 60 μm.

**Figure 5.**
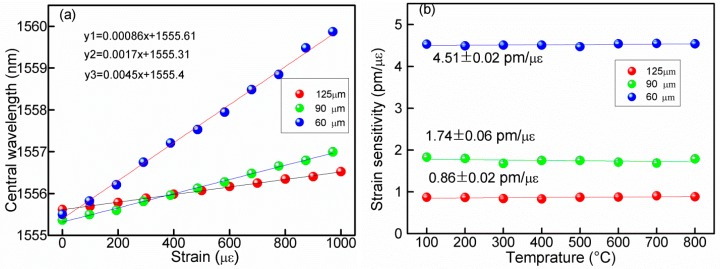
Strain-sensitivity of ER-FBGs with different cladding diameters (**a**) and their stable strain-sensitivity over the temperature from 100 °C to 800 °C (**b**).

To test the strain response of proposed structure, strains are applied to RFBG at each constant temperature. The applied strains on the RFBG are increased from 0 to 1000 με then decreased back to 0 με by steps of 100 uε. At each step, the strain is held 10 min before reading the wavelength. The test results show that no evidence of hysteresis are observed since the highly repeatable and overlapped data are obtained for both increasing and decreasing strains. Figure 4 shows the strain response of RFBGs with different diameters for temperature from 100 °C to 800 °C, the results indicate that the wavelength shifts have good linearity for the applied tensile strain at each constant temperature. Figure 5a indicates that the strain response of RFBGs with different diameters at temperature of 700 °C, and the strain sensitivity is 0.9 pm/με for the RFBG with 125 μm, which is less than the numerical result of 1.2 pm/με of the normal FBGs. This is mainly due to that the different fiber used in our experiment is B/Ge codoped photosensitive fiber including 10 mol% of GeO_2_ and 14–18 mol% of B_2_O_3_. On the other hand, the strain sensitivities of 1.7 pm/με and 4.5 pm/με for ER-FBGs with diameter of 90 μm and 60 μm respectively are obtained at 700 °C, and all the determination coefficients R^2^ are 0.99. Compared with the RFBG with the diameter of 125 μm, the strain sensitivities with diameters of 90 μm and 60 μm are respectively increased around 2.0 and 5.0 times. By reducing the cladding diameter of a FBG through etching with HF, the strain sensitivity can be enhanced significantly. This is mainly due to the effect of stress concentration (geometric discontinuities cause an object to experience a local increase in the intensity of a stress field). The etched fiber section may suffer more stress than that of standard fiber both sides, leading to improved strain sensitivity.

The strain sensitivities are obtained by repeating the above-mentioned experiment many times. The results are shown in Figure 5b, in which the strain sensitivities of ER-FBGs with different diameters at various setting temperatures are 0.9, 1.7 and 4.5 pm/με, corresponding to 125, 90 and 60 μm RFBGs respectively. The results in Figure 5 denote that the strain sensitivity remains constant at each temperature setting. It should be noted that the ER-FBGs operated at high temperature do not reach the glass-softening region and still maintain a reproducible stress response.

## 4. Conclusions

Axial strain responses of ER-FBGs with different diameters have been studied over a large temperature range. The process of chemical etching (from 125 μm to 60 μm) enables the regenerated fiber gratings (at temperature of 680 °C) with a stronger reflective intensity (from 43.7% to 69.8%) together with an improved and linear strain sensitivity (from 0.9 pm/με to 4.5 pm/με) over a large temperature measurement range (from room temperature to 800 °C), making it a good strain sensor candidate for high temperature environments like aerospace engines, and high temperature pipelines in the oil and gas industry.
